# Supplementary material for: Morphological and molecular systematic review of Marphysa Quatrefages, 1865 (Annelida: Eunicidae) species from South Africa
Source: PeerJ. 2020 Oct 21;8:e10076. doi: 10.7717/peerj.10076 (PMC7585384; doi:10.7717/peerj.10076)
Supplement: Data S1 — Results based on Bayesian Inference reconstruction [file peerj-08-10076-s001.docx]

**S1: Species delimitation results: mPTP**

**Results based on Bayesian Inference reconstruction**

Species 1 (support = 1.000)

*Paucibranchia disjuncta* – GQ497549

Species 2 (support = 1.000)

*Hyalinoecia* sp. – GQ497524

Species 3 (support = 1.000)

*Leodice rubra* – GQ497528

Species 4 (support = 1.000)

*Paucibranchia* sp. – JX559753

Species 5 (support = 1.000)

*Paucibranchia bellii* – KT307661

Species 6 (support = 1.000)

*Marphysa regalis* – GQ497562

Species 7 (support = 0.944)

*Marphysa corallina* - KT823410, KT82337, KT823389, KT823343, KT823306, KT823300, KT823271

Species 8 (support = 0.975)

*Marphysa kristiani* - KX172163, KX172162, KX172161, KX172160, KX172159, KX172158, KX172157, KX172156, KX172155, KX172154, KX172153, KX172152, KX172151, KX172150, KX172148, KX172147, KX172145, KX172144, KX172143, KX172142, KX172141, KX172149, KX172146

Species 9 (support = 0.992)

*Marphysa mossambica* - JX559751, KX172164

Species 10 (support = 1.000)

*Marphysa fauchaldi* – KX172165

Species 11 (support = 0.993)

*Marphysa bifurcata* - KX172177, KX172178

Species 12 (support = 0.997)

*Marphysa pseudosessiloa* - KY605405, KY605406

Species 13 (support = 1.000)

*Eunice cf. violaceomaculata* - GQ497542

Species 14 (support = 1.000)

*Palola viridis* – GQ497556

Species 15 (support = 1.000)

*Marphysa californica* - GQ497552

Species 16 (support = 0.978)

***Marphysa sherlockae* n. sp. – XXXXX, XXXXX, XXXXX**

Species 17 (support = 1.000)

*Marphysa brevitentaculata* - GQ497548

Species 18 (support = 1.000)

*Marphysa viridis*_GQ497553

Species 19 (support = 0.944)

*Marphysa victori* - MG384996, MG384999, MG384997, MG384998

Species 20 (support = 0.997)

*Marphysa* sp. – KF733802, NC023124

Species 21 (support = 0.942)

*Marphysa mullawa* - KX172166, KX172167, KX172168, KX172169, KX172170, KX172171, KX172172, KX172173, KX172174, KX172175, KX172176

Species 22 (support = 0.947)

*Marphysa tripectinata* - MN106271, MN10622, MN1062723, MN106274, MN106275,

MN106276, MN106277, MN106278

Species 23 (support = 0.950)

***Marphysa haemasoma -* MN067877, XXXXX, XXXXX, XXXXX, XXXXX**

Species 24 (support = 0.954)

*Marphysa honkongensa –* MH598525, MH598526

Species 25 (support = 0.948)

*Marphysa iloiloensis –* MN133418, MN106279, MN106280, MN106281

Species 26 (support = 0.979)

*Marphysa gaditana* – MN816444, KR916872, KR916873, KP254802, KP254743, KP254643, KP254503, KP254537, KR916871, AY040708, KP255196, KP254890, KP254644, KP254223

Species 27 (support = 0.946)

*Marphysa sanguinea -* GQ497547, MK541904, MK950851, MK950852, MK950853, MK967470, MN106282, MN106283, MN106284

Species 28 (support = 1.000)

*Marphysa* sp. – KR916870

Species 29 (support = 0.924)

*Marphysa aegypti -* MF196969, MF196971, MF196970, MF196968

Species 30 (support = 0.881)

*Marphysa chirigota* - MN816441, MN816442, MN816443
